# Effect of Heat Treatment on the Properties of Wood-Derived Biocarbon Structures

**DOI:** 10.3390/ma11091588

**Published:** 2018-09-02

**Authors:** Min Yu, Theo Saunders, Taicao Su, Francesco Gucci, Michael John Reece

**Affiliations:** 1 School of Engineering and Material Science, Queen Mary University of London, London E1 4NS, UK; min.yu@qmul.ac.uk (M.Y.); t.g.saunders@qmul.ac.uk (T.S.); t.su@qmul.ac.uk (T.S.); f.f.gucci@qmul.ac.uk (F.G.); 2 Nanoforce Technology Limited, London E1 4NS, UK

**Keywords:** graphitization, wood-derived biocarbon, thermal conductivity

## Abstract

Wood-derived porous graphitic biocarbons with hierarchical structures were obtained by high-temperature (2200–2400 °C) non-catalytic graphitization, and their mechanical, electrical and thermal properties are reported for the first time. Compared to amorphous biocarbon produced at 1000 °C, the graphitized biocarbon-2200 °C and biocarbon-2400 °C exhibited increased compressive strength by ~38% (~36 MPa), increased electrical conductivity by ~8 fold (~29 S/cm), and increased thermal conductivity by ~5 fold (~9.5 W/(m·K) at 25 °C). The increase of duration time at 2200 °C contributed to increased thermal conductivity by ~12%, while the increase of temperature from 2200 to 2400 °C did not change their thermal conductivity, indicating that 2200 °C is sufficient for non-catalytic graphitization of wood-derived biocarbon.

## 1. Introduction

Wood-derived biocarbon (biochar, charcoal) structures have gained much attention owing to the hierarchical architecture of their cellular pore structures and the ability to produce complex shapes [1,2,3,4]. The graphitization of carbon has a significant impact on its properties, i.e., the electronic, magnetic and thermal properties [5,6,7]. Graphitic porous biocarbon monoliths are promising because they combine good mechanical properties with low density (0.11–0.97 g/cm^3^) with the properties of graphite (high degree of ordering, low thermal expansion coefficient, good thermal and electrical conductivities) [8]. Two main techniques have been used to graphitize wood-derived biocarbons, including non-catalytic high-temperature (up to 3000 °C) graphitization [5], and low-temperature (1300–1600 °C) catalytic graphitization with Fe, Co, Mn and Ni etc. [8,9,10,11,12]. During the catalytic graphitization process, the catalysts introduce impurities (i.e., carbides, metal particles) into the biocarbon structure, and the graphitic carbon surrounding the catalyst particles (i.e., Fe, Co, and Ni), can be formed at 1000–1600 °C [8,13]. Acid washing (i.e., HNO_3_) is required to remove metal particles in order to achieve pure graphitic carbon. Byrne et al. [14] graphitized wood-derived biocarbon at 2500 °C without the use of a catalyst, however, they did not report their mechanical properties, or electrical and thermal conductivities. Until now, there are few reported works on the effect of temperature and duration time on the properties (especially thermal conductivity) of graphitized wood-derived biocarbon structures prepared by non-catalytic high-temperature (above 2000 °C) graphitization [5].

Porous carbon materials with high thermal conductivity are needed for thermal energy storage, such as thermal enhancers and containers for phase change materials [15,16]. Rico et al. [8] evaluated the thermal conductivity of Fe-catalyst graphitized wood-derived carbon, and found that the thermal diffusivity of graphitized carbon increased with increasing pyrolysis temperatures up to 800 °C, mainly resulting from an increased degree of graphitization. Johnson et al. [17] found that Ni-catalyst graphitized wood-derived carbon has similar properties, and they further infiltrated copper into the pore structures to increase the thermal conductivity. 

In this work, graphitized porous biocarbon monoliths derived from beech wood were obtained by heating at high temperatures (2200–2400 °C) without the use of a catalyst. This heat treatment was performed in a Spark Plasma Sintering (SPS) furnace with high heating and cooling rates (up to 200 °C/min). Accordingly, we report for the first time the effects of temperature and duration time on the properties (compressive strength, electrical and thermal conductivity) of these samples prepared by non-catalytic high temperature graphitization.

## 2. Experimental Process

Cylindrical pieces of beech wood (DOW003100, Tilgear Ltd., Hertfordshire, UK) were chosen as the carbon source. Cylindrical biocarbon structures (Ø = ~6 mm, H = ~9 mm) were prepared by pyrolyzing the beech wood (DOW003100, Tilgear Ltd.) at 1000 °C for 4 h, as performed in our previous work [18]. The prepared biocarbon structures were then heated to higher temperatures (2200 °C and 2400 °C) in Ar for different duration times (2–15 min) in a SPS furnace. A heating rate of 200 °C/min and cooling rate of 100 °C/min were used during this thermal processing. A pressureless mode in SPS was used in order to retain the porous biomorphic structure derived from the wood. The bulk density (geometrical density, which includes pores) of the samples was estimated by dividing the weight by the geometrical volume. The solid density (which excludes the pores) of the samples was measured using the Archimedes’ method. 

An FEI Inspect-F scanning electron microscope (SEM, Hillsboro, OR, USA) was used to characterize the morphology of the samples. Transmission electron microscopy (TEM, JEOL 2010, JEOL, Akishima, Japan) and X-ray diffraction (XRD, Siemens Diffraktometer-D5000, Siemens, Berlin, Germany) analysis with Cu Kα radiation were used to detect the crystalline structures in the samples. Raman spectroscopy (Labspec 6, Horiba Jobin-Yvon, Kyoto, Japan) at room temperature was used to determine the degree of structural disorder in the carbons using an excitation of 514 nm. The degree of crystallinity (β) was calculated using the following Equation (1) [8]:

(1) $$\beta =\frac{I_{G}}{I_{G}+I_{D}}$$

where *I*_G_ and *I*_D_ are the intensities (area under the peak) of the bands G (~1580 cm^−1^) and D (~1350 cm^−1^) in the Raman spectra, respectively. 

The nitrogen absorption-desorption isotherm was measured using an Autosorb-IQ2-MP-C system (Quantachrome Instruments, Boynton Beach, FL, USA). The specific surface area and pore size distribution were calculated using the multipoint Brunauer–Emmett–Teller (BET, Quantachrome Instruments, Boynton Beach, FL, USA) method and Quenched Solid Density Function Theory (QSDFT), respectively.

The compressive strength of a set of six samples with nominal dimensions of Ø = 6 ± 0.1 mm and H = 9 ± 0.3 mm was measured in the axial direction at room temperature using a universal testing device (Model 4202, Instron, Canton, MA, USA). The displacement speed was set at 0.5 mm/min.

The room-temperature electrical conductivity of the samples was measured using a two-point conductivity measurement technique, using a picoameter (Keithley 6485, Keithley, Solon, OH, USA) and DC voltage source (Agilent 6614C, Agilent, Santa Clara, CA, USA).

The thermal diffusivity (*α*) was measured on cylinder samples (diameter: ~6 mm, thickness: ~1.5 mm) using a Netzsch LFA-457 thermal analyzer (Netzsch, Hamburg, Germany). Three measurements were carried out at each temperature in the range of 25–800 °C in a flowing Ar atmosphere. The thermal conductivity (*κ*) was calculated using the following equation: *κ* = *C*_p_ × *D* × *α*. In our work, the specific heat capacity (*C*_p_) of samples was taken from the literature (0.25–2.0 J/(g·K) in the temperature range of 25 to 800 °C [19], and *D* was taken as the bulk density (geometric density). 

## 3. Results and Discussion

**Figure 1:**
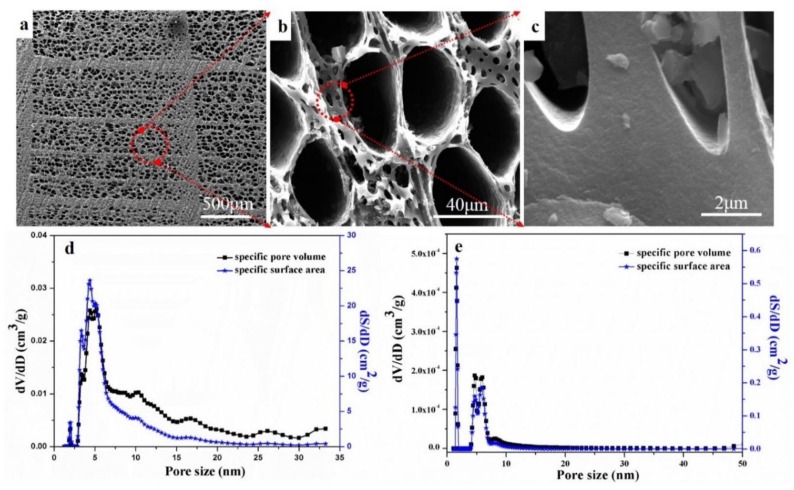
(**a**–**c**) SEM micrographs and (**d**,**e**) pore size distributions (based on BET analysis) of the biocarbon structures obtained at different heat treatment conditions. (**d**) is the sample prepared at 1000 °C for 4 h and (**e**) is the sample prepared at 2400 °C for 10 min.

Figure 1 shows the microstructures and pore size distributions of the wood-derived biocarbons after different heat treatments. The biocarbon-2400 °C exhibited uniform and nearly round macropores with diameters of ~50 μm and ~8 μm, as shown in Figure 1a,b. Dense struts (Figure 1c) were also observed, providing strong mechanical support for the structures. The biocarbon-1000 °C (Figure 1d) exhibited a relatively wide range of micropores (0–25 nm), while the graphitized biocarbon-2400 °C exhibited a micropore distribution mainly concentrated in the range of 0–10 nm (Figure 1e). This might result from the shrinkage of large nano-sized pores (10–50 nm) during the graphitization process. In addition, the specific pore volume and specific surface area of the biocarbon-2400 °C were two orders of magnitude smaller than that of the biocarbon-1000 °C, indicating the disappearance of micropores during the high temperature (2400 °C) treatment. This mainly resulted from the disappearance of small pores (≤50 nm) caused by the rearrangement of carbon structures at high temperatures up to 2400 °C. The shrinkage of nano-sized pores might limit the application of the graphitized biocarbon in the electrochemical energy storage applications.

**Figure 2:**
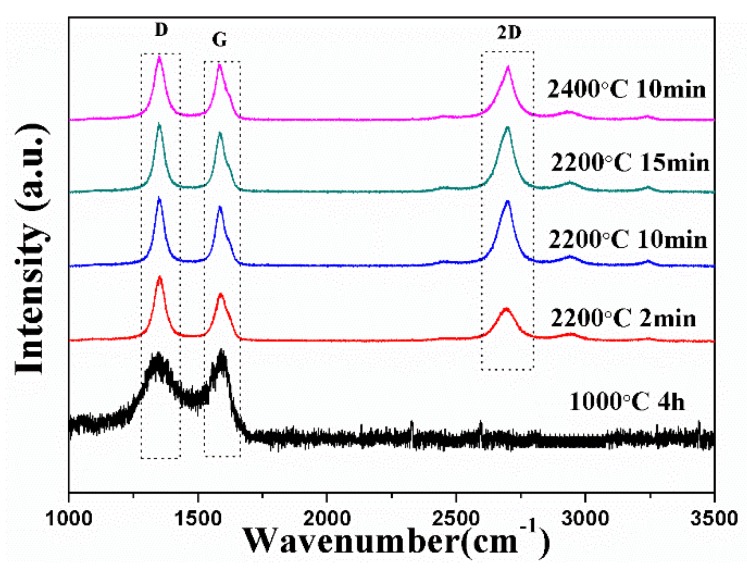
Raman spectra of wood-derived biocarbon prepared at different temperatures and dwell times.

**Figure 3:**
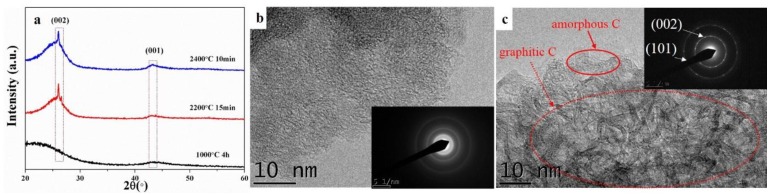
(**a**) XRD patterns of biocarbon structures obtained using different temperatures and dwell times; (**b**,**c**) are high resolution transmission electron microscope (HRTEM) images of the biocarbon structures prepared at 1000 °C and 2400 °C, respectively. The insets are the corresponding selected area electron diffraction (SAED) patterns.

The Raman spectra for the biocarbon-1000 °C exhibited a broad weak D peak at 1360 cm^−1^ and G peak at 1584 cm^−1^, indicating that it contained little graphitic carbon (Figure 2). All of the biocarbons prepared at 2200 °C and 2400 °C exhibited both a sharp D and G peak, which are related to the defect structure of graphite and perfect graphite structure (in-plane stretching of graphite lattice, in-plane vibration of sp^2^ carbon atoms), respectively. The G/D ratio increased in the graphitized biocarbon-2200 °C with the dwell time increasing from 2 to 15 min. This indicates a higher degree of graphitization in the biocarbon, which is further confirmed by the XRD patterns (Figure 3a) and TEM images (Figure 3b,c). The biocarbon-2400 °C exhibited a slightly higher G/D ratio compared to the biocarbon-2200 °C. Both biocarbon-2200 °C and biocarbon-2400 °C exhibited a smaller (~50%) full width at half maximum (FWHM) of their G band compared with biocarbon-1000 °C, indicating a high relative amount of graphitic carbon to amorphous carbon. The corresponding crystalline ratio of the samples was calculated based on the Equation (1), and is shown in Table 1.

The XRD and TEM analysis were also used to further investigate the graphitization of the biocarbons, as shown in Figure 3. The biocarbon-1000 °C exhibited two broad peaks at 2θ = 20–26° and 2θ = 41–46°, which are characteristic of amorphous carbon. Both the biocarbon-2200 °C and biocarbon-2400 °C showed a superposition of two peaks (a broad peak and a sharp peak) at 2θ = 20–28°. Both the biocarbon-2200 °C and biocarbon-2400 °C showed characteristic peaks at 2θ = 26° and 2θ = 43°, which correspond to the reflections of the (002) and (001) planes of graphitic carbon, respectively [20,21], indicating the formation of graphitic carbon, which is in good agreement with the Raman data (Figure 2). The biocarbon-1000 °C exhibited a typical HRTEM image for an amorphous structure (Figure 3b), while the biocarbon-2400 °C showed graphitic carbon layers (see red dashed circle) and some amorphous carbon regions (see red solid circle in Figure 3c). The SAED pattern (inset of Figure 3b) further confirmed the amorphous nature of biocarbon-1000 °C, which is consistent with the XRD data (Figure 3a). The SAED pattern (inset of Figure 3c) further confirmed the crystallinity of the biocarbon-2400 °C, consistent with the peaks in the XRD pattern. 

X-ray photoelectron spectroscopy (XPS) was used to identify c, as shown in Supplementary Figure S1. The XPS survey spectra shown in Figure S1a indicates the presence of C and O in both the biocarbon-1000 °C and biocarbon-2400 °C. The biocarbon-2400 °C exhibited a smaller atomic percentage of O (3.6 at %) than the biocarbon-1000 °C (9.7 at. %). In the high-resolution C 1s spectra (Figure S1b,c), the higher dominant peak at 285.6 eV indicates a higher volume of C=C/C-C in the biocarbon-2400 °C. Both biocarbon-1000 °C and biocarbon-2400 °C exhibited the peaks of C-O and C=O, which are further confirmed in the high resolution O 1s spectra (Supplementary Figure S2).

Table 1 shows the weight loss, density, specific surface area, electrical conductivity, thermal conductivity, crystallinity ratio and compressive strength of the biocarbons. The biocarbon-1000 °C exhibited a bulk density of 0.51 g/cm^3^ and a solid density of 1.85 g/cm^3^. The bulk density of the graphitized biocarbons-2200–2400 °C exhibited a slight decrease (from 0.51 to 0.48 g/cm^3^), owing to a further weight loss of ~10 wt%, probably caused by a mild oxidation and evaporation of the carbon in the SPS chamber during the high temperature graphitization process. However, the solid density of the graphitized biocarbons-2200 and -2400 °C moderately increased to ~2.02 g/cm^3^, owing to the disappearance of nanopores and rearrangement of carbon during the graphitization process at high temperatures (2200–2400 °C). The specific surface area and specific pore volume of graphitized biocarbon-2400 °C compared to the biocarbon-1000 °C decreased from 356 to 144 m^2^/g and from 0.267 to 0.232 cm^3^/g, respectively, owing to the disappearance of micropores (<50 nm) shown in Figure 1e. Compared to biocarbon-1000 °C (~2.8 S/cm), the electrical conductivity of the graphitized biocarbons increased by ten fold (~29 S/cm). This increase was produced by the formation of the graphitic carbon, which is confirmed by the increase of the calculated crystallinity ratio of the graphitized samples given in Table 1. In addition, the compressive strength (36 MPa) of the graphitized samples increased by ~38% compared to biocarbon-1000 °C, again probably resulting from the graphitic carbon formed at 2200–2400 °C. However, the increased duration time from 2 to 15 min and higher temperature from 2200 to 2400 °C, did not significantly increase their compressive strength. 

**Figure 4:**
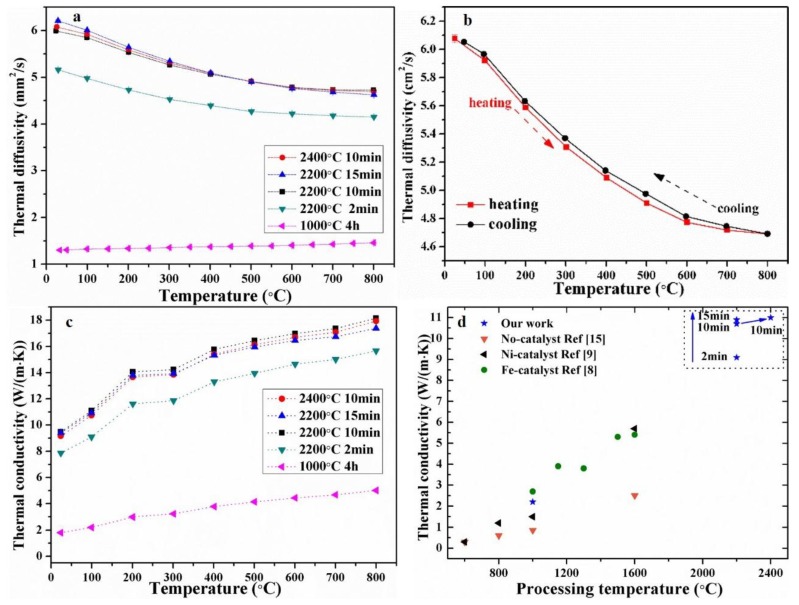
(**a**) Thermal diffusivity as a function of measuring temperatures for biocarbons obtained using different processing temperatures and dwell time; (**b**) Thermal diffusivity versus measuring temperatures during the heating and cooling process of the biocarbon prepared at 2400 °C for 10 min; (**c**) Thermal conductivity as a function of measuring temperatures for biocarbons; (**d**) Comparison of thermal conductivity (at 100 °C) of beech-derived biocarbons prepared using different techniques.

Figure 4 shows the thermal transport properties versus temperatures (25–800 °C) for the wood-derived biocarbon structures prepared at different temperatures (1000–2400 °C) and duration times (2–15 min). As shown in Figure 4a, the measured thermal diffusivity of biocarbon-1000 °C slightly increased with the measuring temperature increasing from 25 to 800 °C. On the contrary, the graphitized biocarbons exhibited decreasing thermal diffusivity with increasing temperature. These thermal diffusivity trends versus measuring temperature are consistent with the reported data for Fe-graphitized biocarbons in the literature [8]. Compared to amorphous biocarbon-1000 °C, the graphitized biocarbons-2200 °C and -2400 °C exhibited much higher thermal diffusivity (up to ~6 mm^2^/s). As shown in Figure 4b, the graphitized biocarbon-2400 °C exhibited similar thermal diffusivity during the heating and cooling process, indicating the stability of the samples during the high-temperature measurements (below 800 °C). 

Figure 4c shows the corresponding thermal conductivity calculated based on the measured diffusivity (Figure 4a) and using values for the heat capacity reported in the literature [15]. All of the samples exhibited increasing thermal conductivity with increasing measuring temperature from 25 to 800 °C. This phenomenon is consistent with the reported results for Fe-graphitized biocarbon structures [8]. The total thermal transfer of the porous biocarbon was mainly through the pores by radiation and struts (pore walls) by electrons and phonons. The contribution of large pores (~1–500 μm) to heat loss by radiation plays a significant role in the thermal transport of porous ceramic foams [22], resulting in the increase of thermal conductivity of biocarbon with increasing measuring temperature. The thermal conductivity of biocarbon-2400 °C is up to 5 times higher than the biocarbon-1000 °C at the same measurement temperature. As polycrystalline graphite has more than two orders higher thermal conductivity than amorphous carbon [15], this higher thermal conductivity of graphitized biocarbon mainly resulted from the formation of graphitic carbon. With the increase of duration time from 2 to 15min at 2200 °C, the thermal conductivity of samples increased moderately by ~12%. This mainly resulted from the increased degree of crystallinity of the biocarbon. The electronic contribution of the thermal conductivity (estimated using Wiedemann-Franz law) in the biocarbon-2400 °C was estimated to be only < ~0.004 W/m/K [8], which is far smaller than the total thermal conductivity (≥ ~2 W/m/K).

Figure 4d shows the comparison of our results with the reported thermal conductivity data for graphitized biocarbon structures derived from beech wood from the literature [8,17]. Our samples exhibited ~89% higher thermal conductivity than the highest result reported in the literature [8,9]. Since the crystallinity ratio of graphitized biocarbon in our work is similar to the reported one for Fe-graphitized biocarbon [8], the high thermal conductivity of our graphitized biocarbon might result from the increased phonon contributions produced by the massively reduced micropore and nanopore volumes (as shown in the BET data in Figure 1). 

## 4. Conclusions

The wood-derived porous monolithic biocarbon structures were graphitized without the use of a catalyst at 2200–2400 °C with high heating and cooling rates (up to 200 °C/min) in a Spark Plasma Sintering (SPS) furnace. The effects of temperatures and duration time on the microstructures of the biocarbons were investigated in detail. Furthermore, the properties of the graphitized biocarbons were also investigated including their mechanical, electrical, and thermal properties. Compared to the un-graphitized biocarbon, the graphitized biocarbons at 2200 °C and 2400 °C exhibited an increased compressive strength of ~38%, and increased room-temperature electrical conductivity of ~8 fold. In addition, the graphitized biocarbons exhibited up to 5 times higher thermal conductivity than the ungraphitized biocarbon. With the increase of duration time from 2 to 15 min at 2200 °C, the graphitized biocarbon exhibited increased thermal conductivity by ~12%, while for the increase of temperature from 2200 to 2400 °C for 10 min, the graphitized biocarbon exhibited similar thermal conductivity. This indicates that 2200 °C might be the optimum temperature for non-catalytic graphitization of wood-derived biocarbon. 

## Figures and Tables

**Table 1:** The weight loss, bulk density, specific surface area, electrical conductivity, thermal conductivity and compressive strength of wood-derived biocarbon prepared at different conditions.

Heat Treatment Condition	Weight Loss (wt %)	Bulk Density (g/cm^3^)	Solid Density (g/cm^3^)	Specific Surface Area (m^2^/g)	Specific Pore Volume (cm^3^/g)	RT Electrical Conductivity (S/cm)	RT Thermal Conductivity (W/(m·K))	Crystallinity Ratio β	Compressive Strength (MPa)
1000 °C, 4 h, Ar	76.6 ± 0.1	0.51 ± 0.02	1.85 ± 0.03	356	0.267	2.8 ± 0.8	1.8	0.25	26 ± 1
2200 °C, 2 min, Ar	86.7 ± 0.1	0.49 ± 0.03	2.03 ± 0.05	-	-	24 ± 0.7	7.8	0.48	35 ± 1
2200 °C, 10 min, Ar	87.5 ± 0.2	0.47 ± 0.02	2.04 ± 0.03	-	-	25 ± 1	9.2	0.48	34 ± 2
2200 °C, 15 min, Ar	85.1 ± 0.1	0.47 ± 0.02	2.03 ± 0.02	-	-	29 ± 0.8	9.4	0.52	36 ± 2
2400 °C, 10 min, Ar	85.2 ± 0.1	0.48 ± 0.04	2.02 ± 0.04	144	0.232	24 ± 0.5	9.5	0.49	36 ± 2

**Note.** Heat treatment conditions refer to the highest temperature and its corresponding duration time, and heating atmosphere. The weight loss is relative to the starting wood.

## Supplementary Materials

The following are available online at http://www.mdpi.com/1996-1944/11/9/1588/s1, Figure S1: (a) Full-scan XPS of biocarbon structures obtained using different temperatures. (b) and (c) are the high resolution C 1s XPS scan of the biocarbon-1000 °C and biocarbon-2400 °C, Figure S2: The high resolution O 1s XPS scan of the biocarbon-1000 °C and biocarbon-2400 °C.
