# Supplementary material for: Effect of Heat Treatment on the Properties of Wood-Derived Biocarbon Structures
Source: Materials (Basel). 2018 Sep 2;11(9):1588. doi: 10.3390/ma11091588 (PMC6163411; doi:10.3390/ma11091588)
Supplement: Supplementary file 1 [file materials-11-01588-s001.pdf]

Supplementary

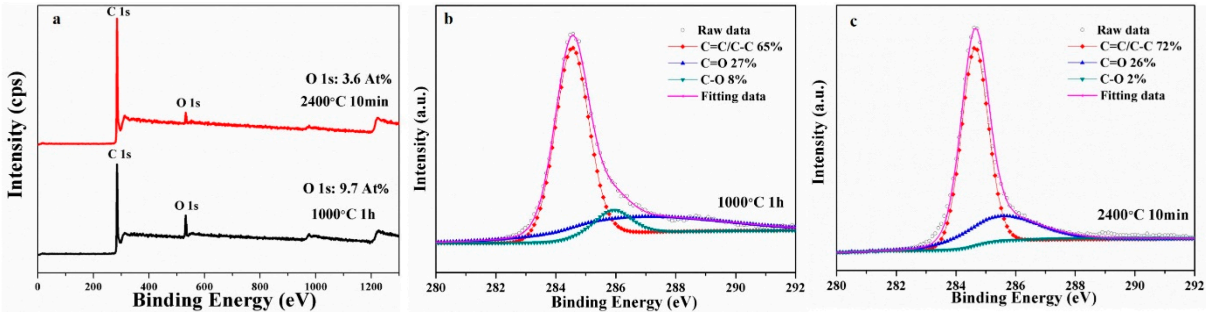

Supplementary Fig. S1. (a) Full-scan XPS of biocarbon structures obtained using different temperatures. (b) and (c) are the high resolution C 1s XPS scan of the biocarbon-1000ºC and biocarbon-2400ºC.

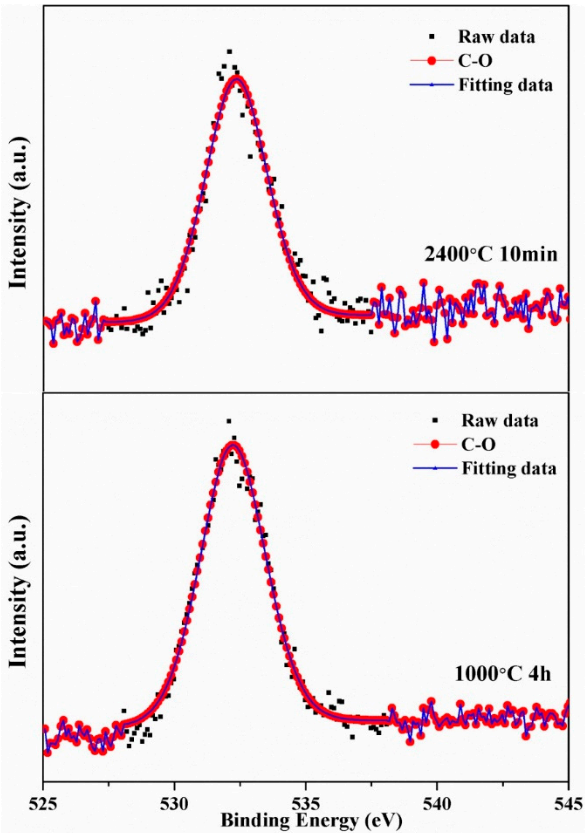

Supplementary Fig. S2. The high resolution O 1s XPS scan of the biocarbon-1000 ºC and biocarbon-2400ºC.
